# Upregulation of Endocan by Epstein-Barr Virus Latent Membrane Protein 1 and Its Clinical Significance in Nasopharyngeal Carcinoma

**DOI:** 10.1371/journal.pone.0082254

**Published:** 2013-12-05

**Authors:** Ping-Hung Yu, Shu-Fan Chou, Chi-Long Chen, Hung Hung, Ching-Yu Lai, Pei-Ming Yang, Yung-Ming Jeng, Shwu-Fang Liaw, Huan-Hsien Kuo, Hey-Chi Hsu, Jen-Yang Chen, Won-Bo Wang

**Affiliations:** 1 Graduate Institute of Microbiology, College of Medicine, National Taiwan University, Taipei, Taiwan; 2 Department of Pathology, Taipei Medial University, Taipei, Taiwan; 3 Graduate Institute of Epidemiology and Preventive Medicine, College of Public Health, National Taiwan University, Taipei, Taiwan; 4 Ph.D. Program for Cancer Biology and Drug Discovery, College of Medical Science and Technology, Taipei Medical University, Taipei, Taiwan; 5 Graduate Institute of Pathology, College of Medicine, National Taiwan University, Taipei, Taiwan; 6 National Institute of Cancer Research, National Health Research Institutes, Zhunan, Taiwan; University of Nebraska - Lincoln, United States of America

## Abstract

Endocan (or called Esm-1) has been shown to have tumorigenic activities and its expression is associated with poor prognosis in various cancers. Latent membrane protein 1 (LMP1) is an Epstein-Barr virus (EBV)-encoded oncoprotein and has been shown to play an important role in the pathogenesis of EBV-associated nasopharyngeal carcinoma (NPC). To further understand the role of LMP1 in the pathogenesis of NPC, microarray analysis of LMP1-regulated genes in epithelial cells was performed. We found that endocan was one of the major cellular genes upregulated by LMP1. This induction of endocan by LMP1 was confirmed in several epithelial cell lines including an NPC cell line. Upregulation of endocan by LMP1 was found to be mediated through the CTAR1 and CTAR2 domains of LMP1 and through the LMP1-activated NF-κB, MEK-ERK and JNK signaling pathways. To study whether endocan was expressed in NPC and whether endocan expression was associated with LMP1 expression in NPC, the expression of endocan and LMP1 in tumor tissues from 42 NPC patients was evaluated by immunohistochemistry. Expression of endocan was found in 52% of NPC specimens. Significant correlation between LMP1 and endocan expression was observed (*p*<0.0001). Moreover, NPC patients with endocan expression were found to have a shorter survival than NPC patients without endocan expression (*p*=0.0104, log-rank test). Univariate and Multivariate analyses revealed that endocan was a potential prognostic factor for NPC. Finally, we demonstrated that endocan could stimulate the migration and invasion ability of endothelial cells and this activity of endocan was dependent on the glycan moiety and the phenylalanine-rich region of endocan. Together, these studies not only identify a new molecular marker that may predict the survival of NPC patients but also provide a new insight to the pathogenesis of NPC.

## Introduction

Nasopharyngeal carcinoma (NPC) is a common cancer in Southern China and Taiwan. The histopathological type of NPC in Southern China and Taiwan is mainly the undifferentiated type, and is closely associated with Epstein-Barr virus (EBV) infection [1]. NPC tumor cells express a limited set of EBV latent proteins including EBV nuclear antigen-1, latent membrane protein (LMP) 1, LMP2A, and LMP2B [2]. Of these proteins, LMP1 has been shown to be an oncoprotein because it is capable of transforming rodent fibroblasts into tumorigenic cells in vitro [3]. Three distinct functional domains of LMP1 have been identified within its C-terminal region: C-terminal activation regions 1, 2, and 3 (CTAR1, CTAR2 and CTAR3). CTAR1 (amino acids 194–231) has been shown to activate the NF-κB pathway through the recruitment of TRAF2 and to activate the pathways of extracellular signal–regulated kinases 1 and 2 (ERK1/2), p38-mitogen-activated protein kinase (p38 MAPK) and phosphoinositide-3 kinase/Akt (PI3K/Akt; [4-7]). CTAR2 (amino acids 351–386) has been shown to activate the NF-κB pathway through the recruitment of TRADD-TRAF2 and to activate the pathways of p38 MAPK and c-Jun N-terminal kinase (JNK) [4,6,8]. CTAR3 (amino acids 232–351), which is located between CTAR1 and CTAR2, directly binds JAK3 resulting in STAT1 phosphorylation and activation [9]. Through activating its downstream signaling pathways, LMP1 has been shown to induce the expression of pro-inflammatory cytokines (IL6 and IL8) [6], angiogenesis factors (VEGF, COX2, RAGE, and HIF-1α) [10-12], various cell surface markers (CD83, CD44, and CD23 etc.) [13-15], anti-apoptotic proteins (A20, Bcl-2, and Bfl-1) [16-18], and proteolytic matrix-degrading enzymes (MMP1 and MMP9) [19,20]. It is thought that LMP1, via activating its downstream signaling pathways and modulating the expression of cellular genes, plays a key role in NPC pathogenesis [21,22]. 

To gain a greater understanding of the role of LMP1 in NPC pathogenesis, a microarray analysis of genes differentially expressed in LMP1-expressing and non-expressing human RHEK-1 epithelial cells was performed. Our microarray data indicated that endocan, a protein that is overexpressed in a variety of human cancers, was greatly upregulated in LMP1-expressing epithelial cells. 

Endocan, also known as endothelial cell-specific molecule-1 (ESM-1), is a soluble proteoglycan of 50 kDa, consisting of a 165 amino-acid core protein and a single dermatan sulfate chain linked to serine 137 residue [23,24]. Endocan was originally cloned from a human endothelial cell cDNA library and is specifically secreted by vascular endothelium in response to pro-inflammatory cytokines and pro-angiogenic factors such as TNF-α, FGF-2, and VEGF [23,25,26]. Endocan is preferentially expressed in tumor endothelium in vivo [25-28]. In certain tumors, endocan is also expressed in tumor cells [29-34]. Together, these data suggest that endocan may play certain roles in tumorigenesis. Indeed, endocan has been shown to have tumorigenic activities because endocan overexpression in nontumorigenic epithelial cells induces tumor formation in *SCID* mice [35]. Functional assays have revealed that endocan inhibits lymphocyte function-associated antigen-1 (LFA-1) and intercellular adhesion molecule-1 (ICAM-1) interaction [36], an important step in the firm adhesion of leukocytes to the endothelium, and thus may regulate the migration of leukocyte into tumor tissues. In addition, endocan can bind the hepatocyte growth factor (HGF)/scatter factor (SF) through its glycan domains and thus promotes the HGF/SF-mediated proliferation of human embryonic kidney cells in a dose-dependent fashion [24]. Recent studies indicate that endocan is one of the genes involved in the switch from dormant to angiogenic tumors [37] and plays an important role in the VEGF-mediated angiogenesis [34,38]. Taken together, these results suggest that endocan, by its roles in modulating cell proliferation, leukocyte function, and angiogenesis, may play an important role in tumor development.

In the present study, we first demonstrated that endocan expression could be induced by LMP1 in various epithelial cells including NPC cells. LMP1 was found to induce endocan expression through its CTAR1 and CTAR2 domains and through the LMP1-mediated NF-κB, MEK-ERK and JNK signaling pathways. Our clinical data indicated that endocan was overexpressed in NPC tissues and its expression was closely associated with LMP1 expression. More importantly, we found that endocan expression was associated with poor prognosis in NPC patients. Finally, we showed that endocan could induce endothelial cell migration and invasion and this ability was dependent on the glycan moiety and the phenylalanine-rich region of endocan.

## Materials and Methods

### Cell culture and reagents

RHEK-1 cells (from Dr. Johng S. Rhim, Laboratory of Cellular and Molecular Biology, National Cancer Institute, USA), a nonmalignant cell line established from normal human foreskin keratinocytes infected with a hybrid virus adenovirus-12-simian virus-40 [39], RHEK-1 derivative cells (constructed in our lab; including RHEK/Tet-LMP1 [40], RHEK/Tet-On [40], LMP135 [41], RHEK-Vec, RHEK-endocan, RHEK-endocan-S21A, RHEK-endocan-F115,116A, and RHEK-endocan-S137A), NPC-TW04 cells, a human NPC cell line from Dr. Chin-Tarng Lin, Department of Pathology, College of Medicine, National Taiwan University [42], and H1299 cells, a human large cell lung carcinoma cell line from American Type Culture Collection (ATCC; Manassas, VA, USA), were cultured in Dulbecco’s modified Eagle’s medium (DMEM) supplemented with 10% fetal bovine serum (FBS), 2 mM L-glutamine and 1% penicillin/streptomycin. Primary human umbilical vein endothelial cells (HUVEC) were isolated from umbilical cord as described [43] and maintained in medium 199 (Invitrogen, Carlsbad, CA, USA) supplemented with 20% FBS, 30 μg/mL endothelial cell growth supplement (Upstate Biotechnology, Lake Placid, NY, USA), 15 µg/mL heparin (Leo Pharmaceutical Product, Ballerup, Denmark), and 1 mM pyruvate. The human microvascular endothelial cell line-1 (HMEC-1, obtained from Centers for Disease Control and Prevention, Atlanta, GA, USA) cells were cultured in MCDB-131 medium (Invitrogen) supplemented with 10% FBS, 2 mM L-glutamine, 1% penicillin/streptomycin, 10 ng/mL human recombinant epidermal growth factor (Becton Dickinson, San Jose, CA, USA), and 1 μg/mL hydrocortisone (Sigma, St Louis, MO, USA). Inhibitors for signaling pathways: BAY11-7082, an inhibitor of IκB kinase; U0126, an inhibitor of ERK1/2; SP600125, an inhibitor of JNK; SB203580, an inhibitor of p38 MAPK; and LY294002, an inhibitor of PI3K, were purchased from Calbiochem (San Diego, CA, USA). All compounds were dissolved in dimethyl sulfoxide (DMSO, Sigma). 

### Plasmids

The plasmids, pIRES-puro-LMP1-386 (encoding wild-type full-length LMP1), pIRES-puro-LMP1Δ189-222 (encoding mutant LMP1 with CTAR1 deleted), pIRES-puro-LMP1-350 (encoding mutant LMP1 with CTAR2 deleted) and pIRES-puro-LMP1-350Δ189-222 (encoding mutant LMP1 with both CTAR1 and 2 deleted) and the vector plasmid pIRES-puro were described previously [44]. The endocan-expressing plasmid pcDNA3.1-endocan was constructed as follows. The endocan cDNA, which was obtained by reverse transcription of HUVEC RNA using oligo dT as a primer, was amplified by polymerase chain reaction (PCR) using the primer pair (forward: 5’-AAAGGTACCCTTCCCACCAGCAAAGACCA-3’; reverse: 5’-AAACTCGAGTCAGCGTGGATTTAACCATT-3’). The PCR product was then cleaved with restriction enzymes *Kpn I* and *Xho I* and cloned into pcDNA3.1 to generate pcDNA3.1-endocan. The mutant endocan-expressing plasmids, pcDNA-endocan-S21A, pcDNA-endocan-F115,116A, and pcDNA-endocan-S137A, were constructed by site-directed mutagenesis using QuikChange® II Site-Directed Mutagenesis Kit (Stratagene, Santa Clara, CA, USA ) and pcDNA3.1-endocan as a template. Primers for S21A mutation were forward: 5’-CCTCTTGCAGCGCGGTGCACTTTTGCA CTCACTGCTGTC-3’; reverse: 5’-GACAGCAGTGAGTGCAAAAGTGCACCGCG CTGCAAGAGG-3’. Primers for F115-116A mutations were forward: 5’-GACTTGGTTACTGAATATTGGGCCGCGGGGAATTTCAGGCATTTTCCCG-3’; reverse: 5’-CGGGAAAATGCCTGAAATTCCCCGCGGCCCAATATTCAGTAAC CAAGTC-3’. Primers for S137A mutation were forward: 5’-CACAATATTGCCATCTCCAGCTGCCATGTCATGC-3’; reverse: 5’-GCATG ACATGGCAGCTGGAGATGGCAATATTGTG-3’. All mutations were verified by DNA sequencing.

### Transient transfection

Plasmid DNA was transfected into NPC-TW04, H1299, or RHEK-1 cells by lipofectamine 2000 transfection reagent (Invitrogen), according to the manufacturer's instructions.

### Establishment of various endocan-expressing RHEK-1 cell clones

pcDNA3.1-endocan, pcDNA-endocan-S21A, pcDNA-endocan-F115,116A, or pcDNA-endocan-S137A was transfected into RHEK-1 cells. After 2 weeks selection with 1000 μg/mL G418, the G418-resistant colonies were cloned and tested for the expression of endocan. Those clones expressing endocan were pooled together and named RHEK-endocan, RHEK-endocan-S21A, RHEK-endocan-F115,116A, and RHEK-endocan-S137A, respectively. The control vector-transfected pooled clone was established by transfecting pcDNA3.1 into RHEK-1 cells followed by selection with 1000 μg/mL G418. The G418-resistant colonies were pooled together and named RHEK-Vec. 

### RNA extraction and cDNA synthesis

Total cellular RNA was extracted from cells using the TRIzol reagent (Invitrogen) according to the manufacturer's instructions. The concentration of RNA was quantified using the NanoDrop ND-1000 Spectrophotometer (Nano-Drop Technologies, Wilmington, DE, USA). The RNA was treated with RNase-free DNase (Invitrogen) before doing reverse transcription to avoid the contamination of DNA. cDNA was synthesized using 1 μg of total RNA as the template and oligo dT as a primer, as described in the manufacturer's protocol for reverse transcription (Invitrogen).

### Real-time RT-PCR

0.05 μg cDNA was used with SYBR Green PCR master mix (Applied Biosystems, Foster City, CA, USA) and sequence-specific primers (see Table S1) in real-time PCR reaction. Amplification of the target sequences was detected with an ABI 7500HT sequence detection system (Applied Biosystems) and analyzed with SDS 2.0 software (Applied Biosystems). The expression level of glyceraldehyde-3-phosphate-dehydrogenase (GAPDH) was used to normalize the abundance of the test transcripts. 

### Semi-quantitative RT-PCR

The generated cDNA was PCR amplified using primer pairs specific for endocan (28 cycles) and GAPDH (18 cycles). The PCR conditions were 30 s at 94 °C, 30 s at 60 °C, and 30 s at 72 °C. The PCR products were loaded and electrophoresed on 2% agarose gel in 1× Tris-acetate-EDTA (TAE) buffer.

### Northern blot Analysis

Northern blot analysis was performed as described previously [45]. Total RNA was isolated from cells with TRIzol reagent (Invitrogen). For Northern blot hybridization, total RNA (20 μg) from each sample was resolved in 1 % denaturing agarose gel and transferred onto to nylon membrane Hybond-N (Amersham, Piscataway, NJ, USA ). Following prehybridization with ULTRAhyb® ultrasensitive hybridization buffer (Ambion, Inc., Austin, TX, USA), the blots were hybridized with P^32^-radiolabeled probe at 42°C. The blots were washed twice in 2 × SSC, 0.1 % sodium dodecyl sulfate (SDS) at 42°C and then washed twice in 0.1 × SSC, 0.1 % SDS at 42°C. The blots were then exposed at -80°C to x-ray film with intensifying screen.

### Western blot analysis

Western blot analyses were performed as described previously [46,47]. Cells were harvested and lysed with ice-cold lysis buffer [50 mM Tris (pH 7.4), 150 mM NaCl, 100 mM NaF, 2 mM Na_3_VO_4_, 1% Triton-X100, 10% glycerol and 1x protease inhibitor cocktail (Roche company, Basel, Switzerland)]. Equivalent amounts of extract protein (20 µg) were resolved in SDS–polyacrylamide gels and electroblotted onto PVDF membranes (Millipore, Billerica, MA, USA). The blots were then incubated with primary antibody followed by incubation with alkaline phosphatase-conjugated secondary antibody. The protein bands were then visualized by using enhanced chemiluminescence reagent (Perkin Elmer Life Sciences, Waltham, MA, USA) followed by autoradiography. For detection of endocan protein in cell supernatants, cells were cultured in serum-free medium. Forty-eight hours later, cell supernatants were concentrated with Amicon Ultra-15 Centrifugal Filter Units (Millipore) to 1/70~1/50 original volume. Equivalent amounts of concentrated samples were resolved in 12% SDS-PAGE and electroblotted onto PVDF membranes. The blots were then incubated with anti-endocan monoclonal antibody (Abnova, Taipei, Taiwan) followed by horseradish peroxidase (HRP) -conjugated secondary antibody.

### Preparation of conditioned medium (CM)

To prepare CM, various endocan-expressing RHEK-1 cells and control cells (1 x 10^6^) were seeded on 10-cm plates. After cells grew to monolayer confluency, cells were washed twice with phosphate-buffered saline (PBS) and incubated in 5 mL serum-free medium for 48 hours. The supernatants were collected and filtered to remove floating cells and cell debris. The filtered CM was store at -80°C until use.

### Measurement of endocan concentrations in the CM by sandwich ELISA

For measurement of endocan concentrations in the CM, sandwich ELISA was performed. ELISA 96-well plates were coated overnight with a goat anti-endocan antibody (10 μg/mL; R&D Systems, Minneapolis, MN, USA) in carbonate buffer (0.1 M Na_2_CO_3_, 0.1 M NaHCO_3_, pH 9.6) at 4 °C. The plates were then washed with wash buffer (PBS containing 0.05% Tween-20) and blocked with 0.5% FBS in PBS at 37°C for 1 h. Next, the wells were incubated with appropriately diluted CM at 37°C for 1 h, followed by incubation with mouse anti-endocan antibody (Abnova) at 37°C for 1 h. Finally, the plates were incubated with anti-mouse secondary antibody conjugated to horseradish peroxidase at 37°C for 1 h and developed with 0.4 mg/mL o-phenylenediamine dihydrochloride (OPD; Sigma) in substrate buffer (0.1 M Na_2_PO4, 0.1 M NaH_2_PO_4_, 0.1 M citric acid, pH 5.5) containing 0.4 μL/mL H_2_O_2_. The color-developing reaction was stopped with 2 N H_2_SO_4_, and the absorbance was measured at 492 nm. A standard curve generated with serial dilutions of recombinant human endocan (from 10 ng/mL to 160 ng/mL) was then used to calculate the concentration of endocan in the CM.

### Antibodies

Mouse monoclonal antibody against LMP1, CS1-4, was from DAKO (Glostrup, Denmark). Mouse monoclonal antibody against endocan was from Abnova. Mouse monoclonal antibody against GAPDH was from Biodesign Inc. (Saco, ME, USA). Mouse monoclonal antibody against phosphorylated IκBα was from Cell Signaling Techonology (Beverly, MA, USA). Rabbit polyclonal antibodies against ERK1/2, phosphorylated ERK1/2, c-Jun, phosphorylated c-Jun, phosphorylated ATF-2, Akt, and phosphorylated Akt were from Cell Signaling Technology. Rabbit polyclonal antibody against ATF-2 was from Santa Cruz Biotechnology, Inc. (Santa Cruz, CA, USA). Goat polyclonal antibody against endocan was from R&D Systems. 

### Endothelial cell transmigration and invasion assays

The in vitro endothelial cell migration and invasion assays were performed as described [48], by using a 24-well Transwell Boyden Chamber (Millipore). For endothelial cell invasion assays, the filter membranes of the Boyden Chamber were pre-coated with Matrigel (30 µg, Becton Dickinson). Briefly, the HMEC-1 or HUVEC cells suspended in 200 µl serum-free MCDB-131 or 199 medium (Invitrogen), respectively, were seeded onto the upper compartment of the transwell chamber. The lower compartment was filled with serum-free CM from various endocan-expressing RHEK-1 clones or control cells. After incubation for 16 h (for migration assays) or 48 h (for invasion assays), the cells migrated to the lower chamber were fixed and stained as described [47,48]. The migrated cells were visualized and counted from six randomly selected fields under inverted microscope. For antibody treated groups, the CM was incubated with control or anti-endocan monoclonal antibody (Abnova; 200 ng/mL) before added to the lower chamber. 

### Patients and tissue specimens

This study was conducted on paraffin-embedded NPC samples from 42 NPC patients who were histologically and clinically diagnosed in the National Taiwan University Hospital between 1997 and 2001. The 42 patients included 32 male and 10 female with a mean age of 47.3 years (range, 15~78 years). The tumors were classified in accordance to the WHO classification system. WHO type I is keratinizing squamous cell carcinoma, type II is non-keratinizing differentiated carcinoma, and type III is non-keratinizing undifferentiated carcinoma. In this study, the NPC samples were composed of 9 non-keratinizing differentiated carcinoma (WHO type II) and 33 non-keratinizing undifferentiated carcinoma (WHO type III). Written consents for the use of these NPC specimens for research purposes have been obtained from the patients or the patient’s parent (in the case of 15 year-old patient). This study has been approved by the National Taiwan University Hospital Research Ethics Committee. 

### Immunohistochemical staining

The protocol used was described previously [49]. Consecutive sections of the NPC tumors were stained for LMP1 and endocan. The following antibodies were used as primary antibodies: mouse monoclonal antibody against LMP1 was from DAKO and mouse monoclonal antibody against endocan was from Abnova. The sections were color-developed with Super Sensitive Polymer-HRP IHC Detection System (BioGenex, San Ramon, CA, USA). The sections were counterstained with hematoxylin.

### Evaluation of immunohistochemical staining for LMP1 and endocan proteins

The stained sections were independently examined by two of the authors (P-H Y and C-L C). Arbitrarily chosen microscopic fields (200×) were evaluated for immunoreactive cells. After counting both immunoreactive cells and the total number of tumor cells, the average percentages of immunoreactive cells were calculated. The results for each protein were classified into four scores, depending on the percentage of immunoreactive tumor cells: 0, < 10 %; 1, 10% -25%; 2, 26%-50%; 3, > 50%. 

### Statistical analysis

Statistical differences between two groups were determined by student’s *t* test. Correlations between LMP1 and endocan protein expression were analyzed by χ^2^ test. Survival analysis was performed by the Kaplan-Meier method. Kaplan-Meier curves were plotted for NPC patients stratified by the endocan or LMP1 status, and the log-rank test was implemented to test the difference of two curves. To further study the effect of endocan on the survival time after adjusting for various clinicopathological factors (age, gender, histologic classification, distant metastasis and endocan), we fit univariate and multivariate Cox proportional hazard model on these potential factors. Statistical analyses were done by SPSS software. A *p*-value of <0.05 is considered statistically significant.

## Results

### Microarray analysis of genes differentially regulated by EBV LMP1 in epithelial cells

**Figure 1:**
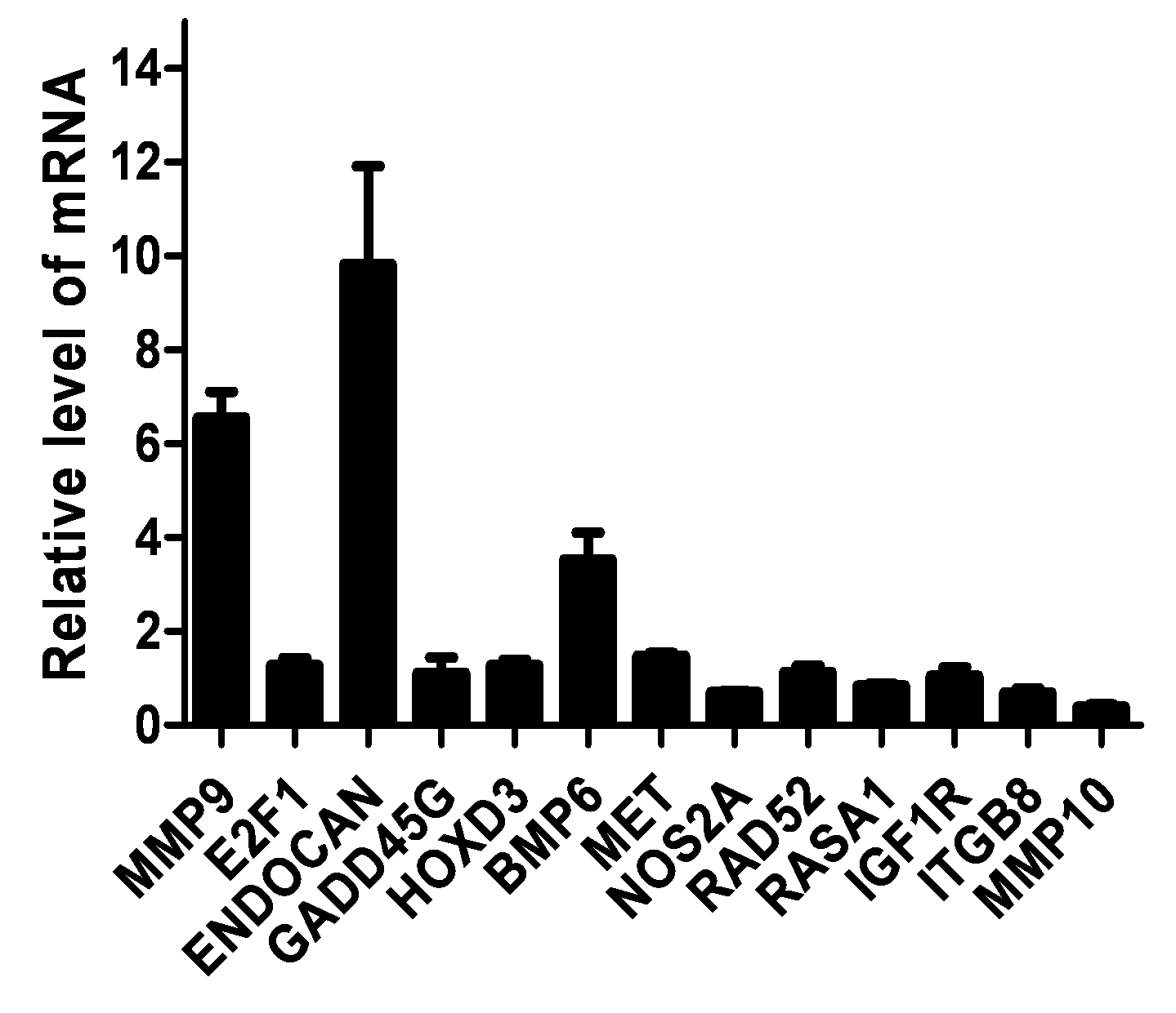
Validation of LMP1-regulated genes by quantitative real-time RT-PCR. Total RNA from doxycycline-treated and untreated RHEK/Tet-LMP1 cells was extracted and subjected to quantitative real-time RT-PCR. The level of respective mRNA expressed in the untreated RHEK/Tet-LMP1 cells was set as 1. Values represent means ± SD.

EBV LMP1 protein has been shown to play a key role in NPC pathogenesis [21,22]. To gain a further understanding of the role of LMP1 in the pathogenesis of NPC, microarray analysis of LMP1-regulated genes in epithelial cells was performed. The RHEK-1-derived cell line, RHEK/Tet-LMP1, which was previously constructed in our laboratory using a Tet-On expression system [40], was used. The expression of LMP1 in the RHEK/Tet-LMP1 cells can be induced by doxycycline, an analogue of tetracycline (data not shown). Total RNA was extracted from doxycycline-treated and untreated RHEK/Tet-LMP1 cells and was subjected to two-color microarray analysis (see Methods S1). Comparison of gene expression between doxycycline-treated and untreated RHEK/Tet-LMP1 cells was performed in duplicate. Genes that are upregulated (> 4-fold) or downregulated (≦ 4-fold) by LMP1 are listed in Table S2. A total of 60 genes were differentially expressed in the doxycycline-treated RHEK/Tet-LMP1 cells compared to the untreated RHEK/Tet-LMP1 cells. Of these LMP1-regulated genes, 42 genes were upregulated, whereas 18 genes were downregulated. Seven upregulated and 6 downregulated genes in Table S2 were selected and tested by quantitative real-time RT-PCR. The upregulation of MMP9, endocan, and BMP6, as well as the downregulation of MMP10 was confirmed in doxycycline-treated RHEK/Tet-LMP1 cells (> 2-fold difference) (Figure 1). The upregulation or downregulation of these genes is indeed due to the effect of LMP1 but not to the effect of doxycycline, because addition of doxycycline to parental RHEK-1 cells did not alter the expression of these genes (data not shown). It is noted that, among the LMP1-regulated genes, endocan was induced at highest level (about 10-fold) by LMP1 (Figure 1). 

### LMP1 induces endocan expression in various epithelial cells

**Figure 2:**
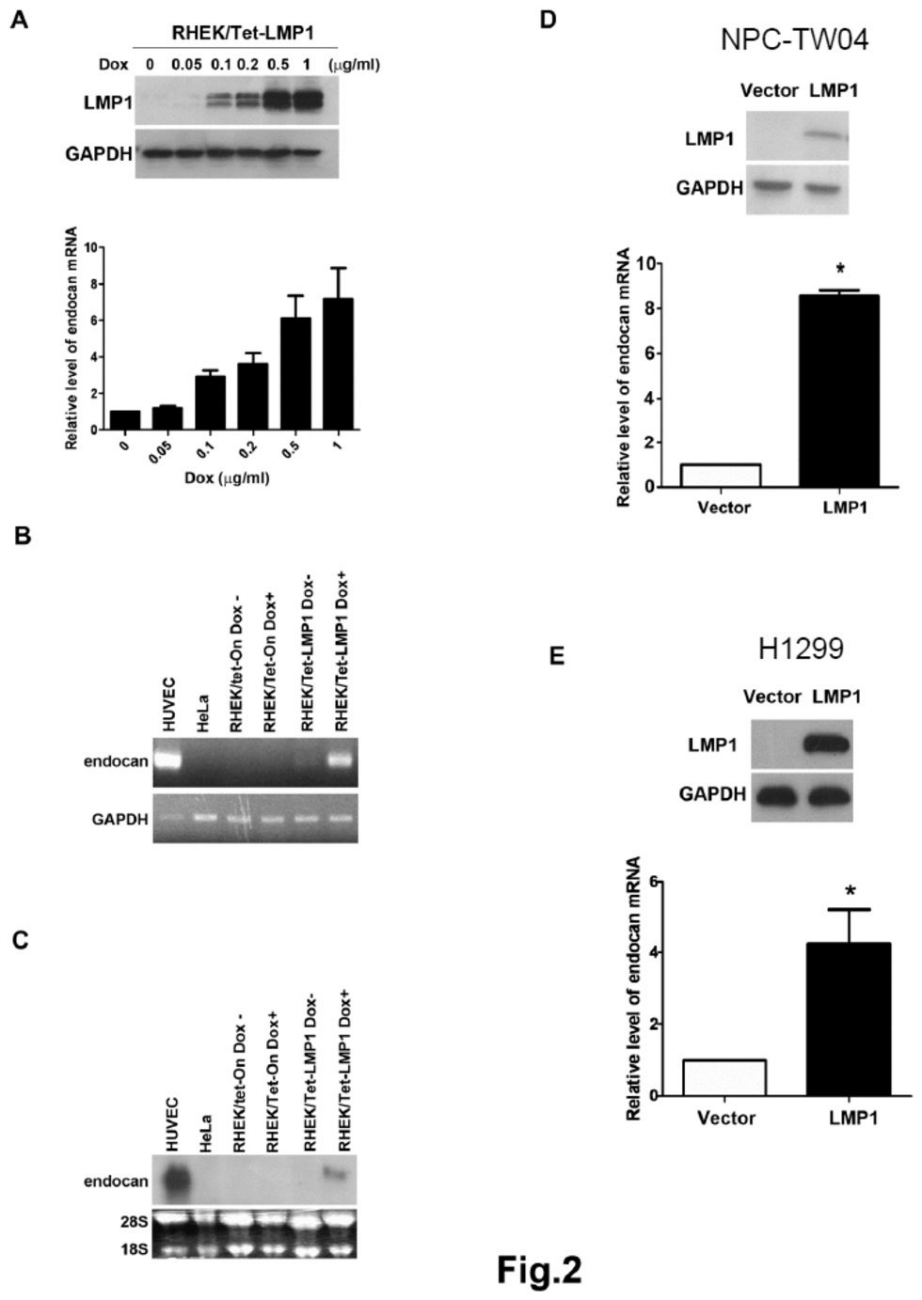
Induction of endocan expression by LMP1 in epithelial cell lines. (**A**) RHEK/Tet-LMP1 cells were incubated with indicated concentrations of doxycycline (Dox) for 36 h. The expression of LMP1 protein and endocan mRNA was analyzed by Western blot analysis and quantitative real-time RT-PCR, respectively. The upper panel shows the expression of LMP1 protein. GAPDH serves as an internal control for amounts of protein loaded on the gel. The bottom panel shows the relative level of endocan mRNA. The level of endocan mRNA expressed in RHEK/Tet-LMP1 cells in the absence of doxycycline was set as 1. Values represent means ± SD. (**B**) RHEK/Tet-On and RHEK/Tet-LMP1 cells were incubated without or with doxycycline (1 μg/mL) for 36 h. Semi-quantitative RT-PCR was performed to determine the expression level of endocan mRNA. The expression of endocan mRNA in HUVEC (which expresses high level of endocan) and HeLa cells (which do not express endocan) were also analyzed. GAPDH serves as an internal control for amounts of RNA used in the assay. (**C**) RHEK/Tet-On and RHEK/Tet-LMP1 cells were treated as in B. Northern blot analysis using 20 μg of total RNA was performed to determine the expression level of endocan mRNA. 28S and 18S rRNA were used as internal controls for amounts of RNA loaded on the gel. (**D** and **E**) NPC-TW04 (**D**) and H1299 (**E**) cells were transiently transfected with 1 µg of pIRES-puro (vector) or pIRES-puro-LMP1-386 (LMP1-expressing plasmid) and incubated for 36 h. The expression of LMP1 protein and endocan mRNA was analyzed by Western blot analysis and quantitative real-time RT-PCR, respectively. The upper panel shows the expression of LMP1 protein. The lower panel shows the relative level of endocan mRNA. The level of endocan mRNA expressed in vector-transfected cells was set as 1. Values represent means ± SD. * *P* < 0.05 versus vector-transfected cells.

Our quantitative real-time RT-PCR data indicate that endocan is one of the major genes induced by LMP1 in RHEK-1 epithelial cells. To further study the induction of endocan by LMP1, we measured the expression of endocan mRNA in RHEK/Tet-LMP1 cells in the presence of various concentrations of doxycycline. The expression of LMP1 in the RHEK/Tet-LMP1 cells was induced by doxycycline in a dose-dependent manner (Figure 2A, upper panel). In parallel, the expression of endocan mRNA determined by real-time RT-PCR was also induced by doxycycline in a dose-dependent manner (Figure 2A, lower panel). These data indicate that LMP1 can induce endocan expression in the RHEK-1 epithelial cells. To further confirm this, the expression of endocan mRNA was measured by semi-quantitative RT-PCR (Figure 2B) and Northern blot analysis (Figure 2C). In consistence with the above data, the expression of endocan was significantly increased in RHEK/Tet-LMP1 cells upon doxycycline addition. In contrast, in RHEK/Tet-On cells, a vector control cell line for RHEK/Tet-LMP1 cells, the addition of doxycycline did not affect the expression of endocan mRNA. Together, these data demonstrate that endocan expression is induced by LMP1 but not by doxycycline in the RHEK/Tet-LMP1 epithelial cells. 

To study whether LMP1 could induce endocan expression in other epithelial cells, NPC-TW04 (a human NPC cell line) and H1299 (a human large cell lung carcinoma cell line) cells were used in the transient transfection assays. Cells were either transfected with the vector (pIRES-puro) or LMP1-expressing plasmid (pIRES-puro-LMP1-386), and the level of endocan mRNA was measured by real-time RT-PCR. Our data indicated that endocan mRNA was significantly induced in LMP1-transfected NPC-TW04 (Figure 2D) and H1299 (Figure 2E) cells. 

### LMP1 induces endocan expression through the CTAR1 and CTAR2 domain

**Figure 3:**
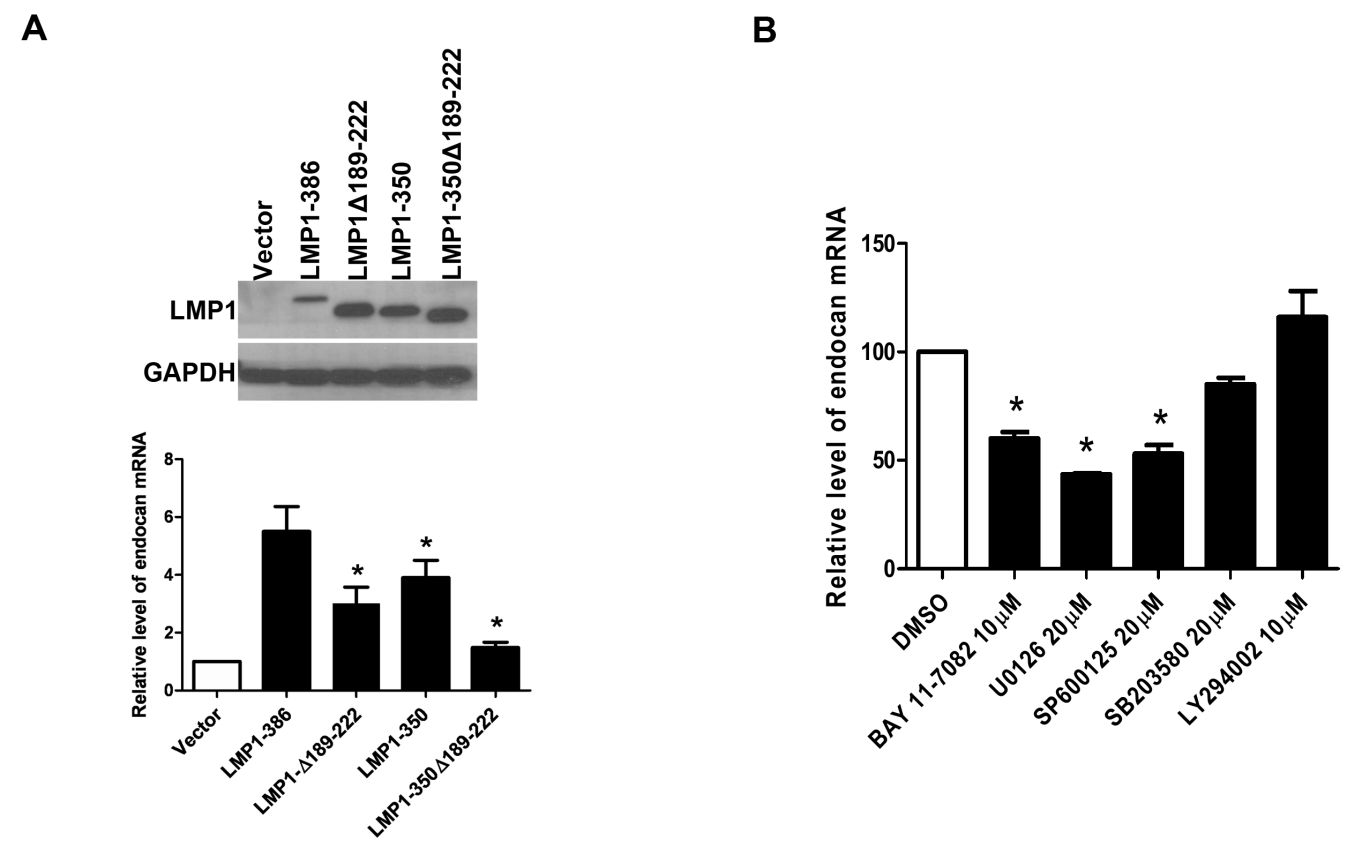
The signaling pathways involved in LMP1 induction of endocan. (**A**) Upregulation of endocan expression by LMP1 is mediated through the CTAR1 and CTAR2 domains of LMP1. 2 x 10^5^ H1299 cells were transfected with 1 μg of pIRES-puro-LMP1-386, pIRES-puro-LMP1Δ189-222, pIRES-puro-LMP1-350, pIRES-puro-LMP1-350Δ189-222 or vector plasmid (pIRES-puro). 24 h later, the expression of the full-length and mutant LMP1 proteins were determined by Western blot analysis (upper panel) and the expression of endocan mRNA was determined by quantitative real-time RT-PCR. The level of endocan mRNA expressed in cells transfected with vector plasmid was set as 1 and the relative levels of endocan mRNA expressed in different transfected cells are shown in lower panel. Values represent means ± SD. * *P* < 0.05 versus pIRES-puro-LMP1-386-transfected cells. (**B**) Upregulation of endocan expression by LMP1 is mediated through the NF-κB, MEK-ERK and JNK signaling pathways. RHEK/Tet-LMP1 cells were pretreated for 1 h with DMSO (solvent for inhibitors) or various signaling pathway inhibitors as indicated. Cells were then incubated in media containing 1 μg/mL doxycycline for 36 h. Total RNA was extracted and the amount of endocan mRNA expressed in the presence of various inhibitors was quantitated by quantitative real-time RT-PCR. The level of endocan mRNA expressed in cells treated with DMSO was set as 100%. Values represent means ± SD. * *P* < 0.05 versus DMSO-treated cells.

LMP1 is known to activate distinct signaling pathways through the CTAR1 or CTAR2 domain. To map the functional domains of LMP1 involved in endocan induction, LMP1 deletion mutants, including LMPΔ189-222 (CTAR1 is deleted), LMP1-350 (CTAR2 is deleted), and LMP1-350Δ189-222 (both CTAR1 and CTAR2 are deleted), were transiently transfected into H1299 cells (The transfection efficiency in H1299 cells is much higher than that in RHEK-1 cells). The expression levels of LMP1 protein in wild-type LMP1 (LMP1-386) and mutant LMP1-transfected cells were analyzed by Western blot analysis (Figure 3A, upper panel). The levels of endocan mRNA expressed in transfected cells were measured by real-time RT-PCR. As shown in Figure 3A (lower panel), while the wild-type LMP1 (LMP1-386) dramatically induced the expression of endocan, the ability of the CTAR1 deletion mutant to induce endocan expression was significantly reduced. The endocan-inducing ability of the CTAR2 deletion mutant was also impaired but to a less extent. When both CTAR1 and CTAR2 are deleted, the mutant barely had an ability to induce endocan expression. Together, these data clearly indicate that CTAR1 and CTAR2 are involved in LMP1-mediated endocan induction. 

### LMP1 acts via the NF-κB, MEK-ERK, and JNK pathways to induce endocan

Previous studies have shown that LMP1 can activate multiple signaling pathways, including NF-κB, MEK-ERK, p38 MAPK, PI3K-Akt, and JNK pathways, in the cells. To study which signaling pathways are involved in LMP1 induction of endocan, the effects of specific inhibitors of the above signaling pathways on the expression of endocan in RHEK/Tet-LMP1 cells were tested. Following treatment with inhibitor for NF-κB (BAY11-7082), MEK-ERK (U0126), JNK (SP600125), p38 MAPK (SB203580), or PI3K-Akt (LY294002) pathway, the RHEK/Tet-LMP1 cells were incubated in the doxycycline-containing medium to induce the expression of LMP1. The expression of endocan mRNA in the presence or absence of specific inhibitors was then determined by real-time RT-PCR. Our data indicated that endocan mRNA expression was significantly reduced by BAY11-7082, U0126, and SP600125 but not by SB203580 and LY294002 (Figure 3B). These inhibitors did not affect the expression of LMP1 in doxycycline-treated RHEK/Tet-LMP1 cells (Figure S1). We also showed that LMP1 could activate the NF-κB, MEK-ERK, JNK, p38 MAPK, and PI3K-Akt signaling pathways in the cells and that the inhibitors of these pathways were effective in inhibiting the respective signaling pathways (Figure S1). Together, these data demonstrate that LMP1 upregulates endocan expression through the NF-κB, MEK-ERK, and JNK signaling pathways.

### Endocan is overexpressed in NPC tissues and its expression is correlated with expression of LMP1 protein

**Figure 4:**
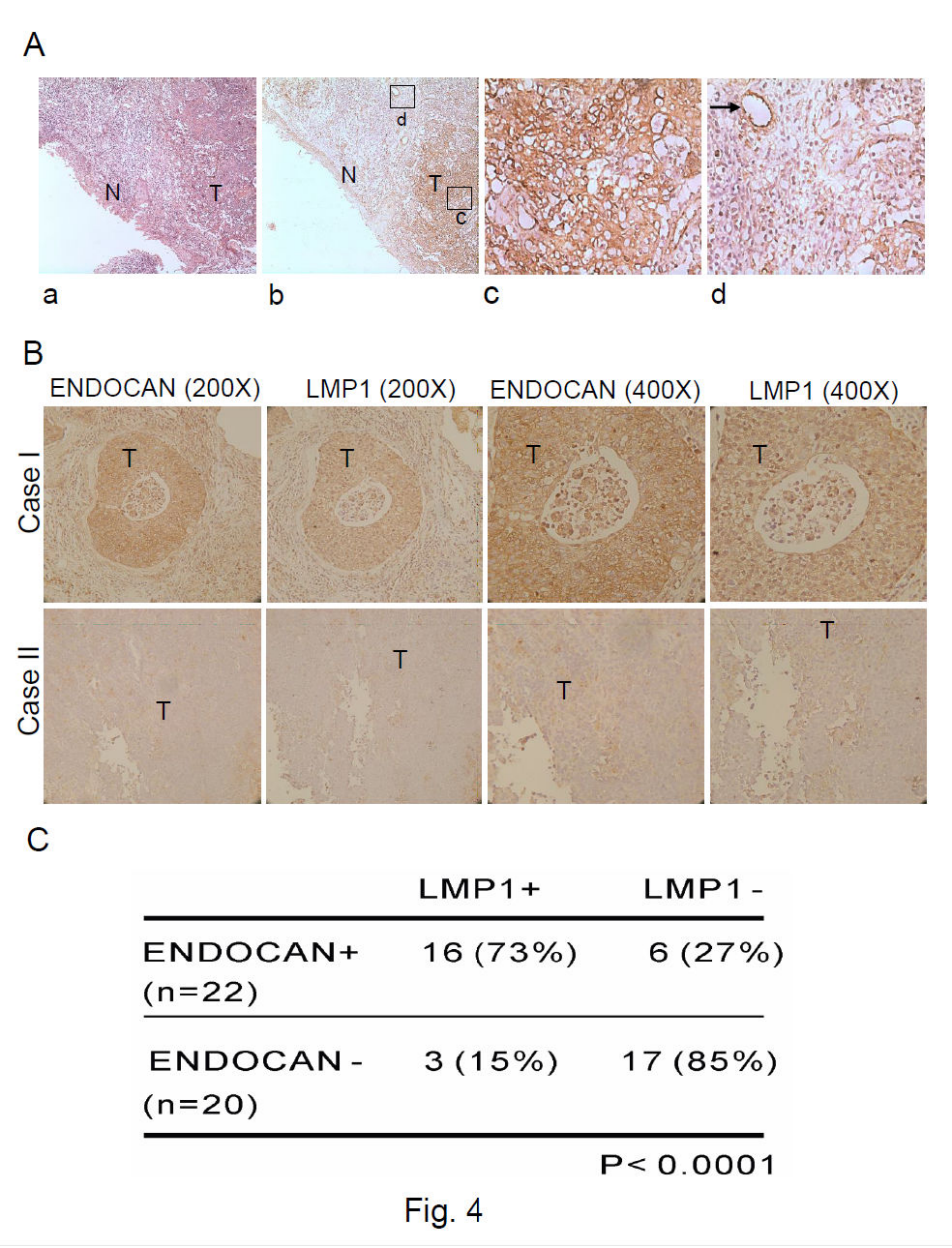
Endocan is overexpressed in NPC tissues and its expression is correlated with LMP1 expression. (**A**) Immunohistochemical staining of endocan in NPC tissues. Panel a, H&E staining (original magnification, ×100). Panel b, Endocan was expressed in NPC tumor cells but not in adjacent normal epithelial cells (original magnification, ×100). Panel c, The tumor part (marked as square c) in Panel b was photographed at higher magnification (original magnification, ×400). Panel d, Square d in Panel b was photographed at higher magnification (original magnification, ×400). Endocan expression was found in vascular endothelial cells adjacent to the tumor. Arrowhead indicates vascular endothelial cells. T, tumor cells; N, normal epithelial cells. The brown color represents endocan staining. (**B**) Immunohistochemical staining of endocan and LMP1 in NPC tissues. Case I shows the representative result with high endocan and LMP1 expression in consecutive sections. Case II shows the representative result with negative staining of endocan and LMP1 in consecutive sections. The brown color represents endocan or LMP1 staining. T, tumor cells. (**C**) Association between endocan and LMP1 protein expressions in 42 NPC cases. Statistical relationships between endocan and LMP1 expressions were analyzed by χ^2^ test.

Knowing that endocan is upregulated by LMP1 and that LMP1 is expressed in 20 to 65 % of NPC tissues [50], we thus tested whether endocan was overexpressed in NPC and if yes, whether the expression of endocan was correlated with that of LMP1 in NPC tissues. To do this, expression of endocan and LMP1 was examined in the consecutive sections of NPC tumors from 42 patients by immunohistochemical staining. The representative results of endocan immunostaining were shown in Figure 4A. Endocan was detected in the cytoplasm and cell membrane of NPC tumor cells, whereas no endocan expression was observed in the adjacent normal epithelial cells (Figure 4A, panel b and c). Endocan is known to be expressed in tumor endothelium (27). To confirm that endocan is expressed in the NPC cells rather than the endothelial cells in the NPC specimens, the endocan-positive NPC tissue was stained with antibody against von Willebrand factor (vWF), a marker for endothelial cells. As shown in Figure S2, the NPC tissue was largely stained vWF-negative, indicating that the cells expressing endocan are NPC cells rather than endothelial cells. It has been reported that endothelial cells in the vicinity of the tumor can be induced to express endocan by tumor-derived factors (27). In consistence with this, we also noted that endocan was expressed in the vascular endothelial cells adjacent to tumors (Figure 4A, panel d and Figure S3). Overall, the positive immunostaining rate of endocan expression in NPC was 52% (Table 1). This is the first study showing that endocan is overexpressed in the NPC tissues. 

**Table 1:** Summary of immunohistochemical staining results for endocan and LMP1 in 42 NPC cases.

Patient no.	Endocan	LMP1	Patient no.	Endocan	LMP1
1	1	1	22	3	3
2	2	1	23	3	0
3	0	0	24	0	0
4	3	3	25	2	2
5	0	0	26	3	1
6	0	1	27	0	0
7	0	0	28	0	1
8	3	2	29	0	0
9	3	3	30	2	2
10	0	0	31	3	3
11	3	0	32	2	2
12	0	0	33	0	0
13	3	1	34	3	1
14	0	0	35	3	1
15	2	2	36	2	0
16	1	0	37	0	0
17	0	0	38	1	1
18	1	0	39	0	0
19	3	0	40	0	0
20	0	0	41	0	3
21	0	0	42	0	0

NOTE: The results for each protein were classified into four scores, depending on the percentage of immunoreactive tumor cells: 0, < 10%; 1, 10% -25%; 2, 26%-50%; 3, > 50%.

The representative staining results of endocan and LMP1 in the consecutive sections of the endocan-positive or -negative NPC tumor were shown in Figure 4B. LMP1 was detected in the cell membrane of NPC tumor cells, and was detected in 19 of 42 NPC tissues (45%) (Table 1). Among these 19 LMP1-positive NPC tumors, 16 of them also expressed endocan (Table 1). Statistical relationship between endocan and LMP1 expressions was analyzed by χ^2^ test. The data showed that the expression of these two proteins in NPC tissues was significantly correlated (*p* < 0.0001) (Figure 4C).

### Relationship between endocan expression and survival in NPC patients

**Figure 5:**
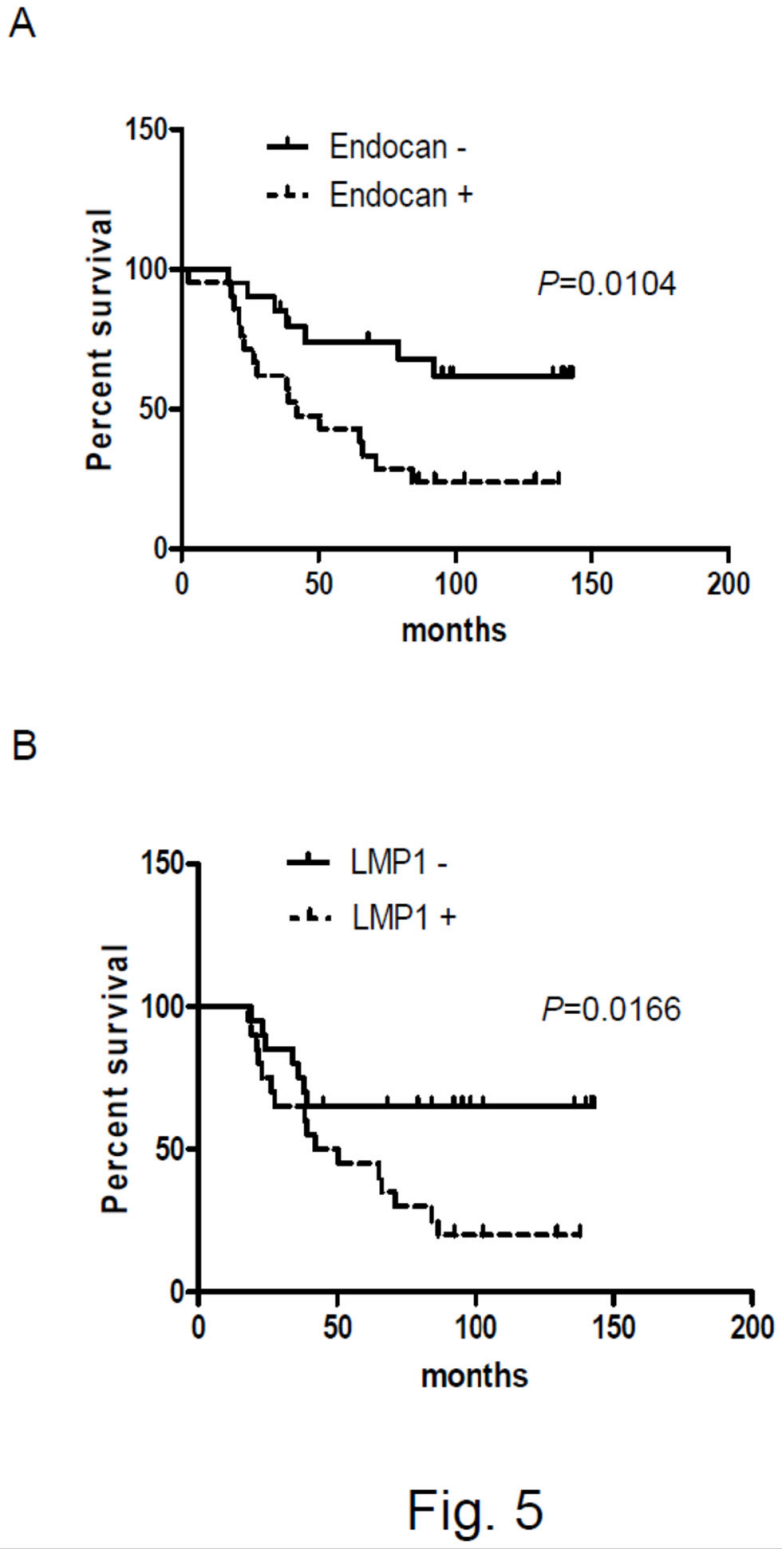
Relationship between survival and endocan expression or LMP1 expression in NPC patients. (**A**) Overall survival curve of endocan-positive (n=21; dotted line) and endocan-negative (n=20; solid line) NPC patients. A statistically significant difference was found between these two groups of patients (*P* = 0.0104, log-rank test). (**B**) Overall survival curve of LMP1-positive (n=19; dotted line) and LMP1-negative (n=22; solid line) NPC patients. A statistically significant difference was found between these two groups of patients (*P* = 0.0166, log-rank test).

Endocan has been shown to be an important prognostic factor in several cancers [25,28,31,34,51]. We thus analyzed the relationship between endocan expression and survival in NPC patients. We divided the NPC cases into 2 groups based on the expression of endocan in tumor cells: endocan-positive (endocan expressed in ≧ 10% of tumor cells) and endocan-negative (endocan expressed in < 10% of tumor cells). As shown in Figure 5A, the endocan-positive NPC patient group had a shorter survival than the endocan-negative NPC patient group (*p* = 0.0104, log-rank test). We also analyzed the relationship between LMP1 expression and survival in NPC patients. As shown in Figure 5B, the LMP1-positive NPC (LMP1 expressed in ≧ 10% of tumor cells) patient group had a shorter survival than the LMP1-negative NPC (LMP1 expressed in < 10% of tumor cells) patient group (*p* = 0.0166, log-rank test).

### Prognostic value of endocan in patients with NPC

To determine if endocan expression level is an independent prognostic factor for NPC, we fit univariate and multivariate Cox proportional hazard model on endocan expression level, age, gender, histologic classification and distant metastasis (Table 2). In both analyses, endocan expression was recognized as having significant effect (*p* < 0.05), which indicates that endocan expression level is a potential prognostic factor for NPC.

**Table 2:** Univariate and multivariate analyses of factors associated with survival in patients with NPC.

**Characteristics**		**Univariate analysis**			**Multivariate analysis**	
	**No. patients**	***P***	**Harzard ratio**(95% CI^**^)		***P***	**Hazard ratio(95% CI)**
Age^*^	36	0.046	1.038 (1.001-1.076)		0.057	1.041 (0.999-1.086)
Sex						
Male	28	0.778	1.171 (0.391-3.506)		0.563	1.401 (0.446-4.398)
Female	8					
Histologic classification						
Type II	9	0.150	0.405 (0.118-1.388)		0.107	0.309 (0.075-1.289)
Type III	27					
Distant metastasis						
Presence	8	0.008	3.566 (1.386-9.176)		0.118	2.264 (0.813-6.307)
Absence	28					
Endocan						
Positive	20	0.012	3.683 (1.327-10.216)		0.036	3.140 (1.078-9.142)
Negative	16					

^*^ Age was treated as a continuous variable

^**^ CI, confidence interval

### Endocan stimulates the migration and invasion ability of endothelial cells

**Figure 6:**
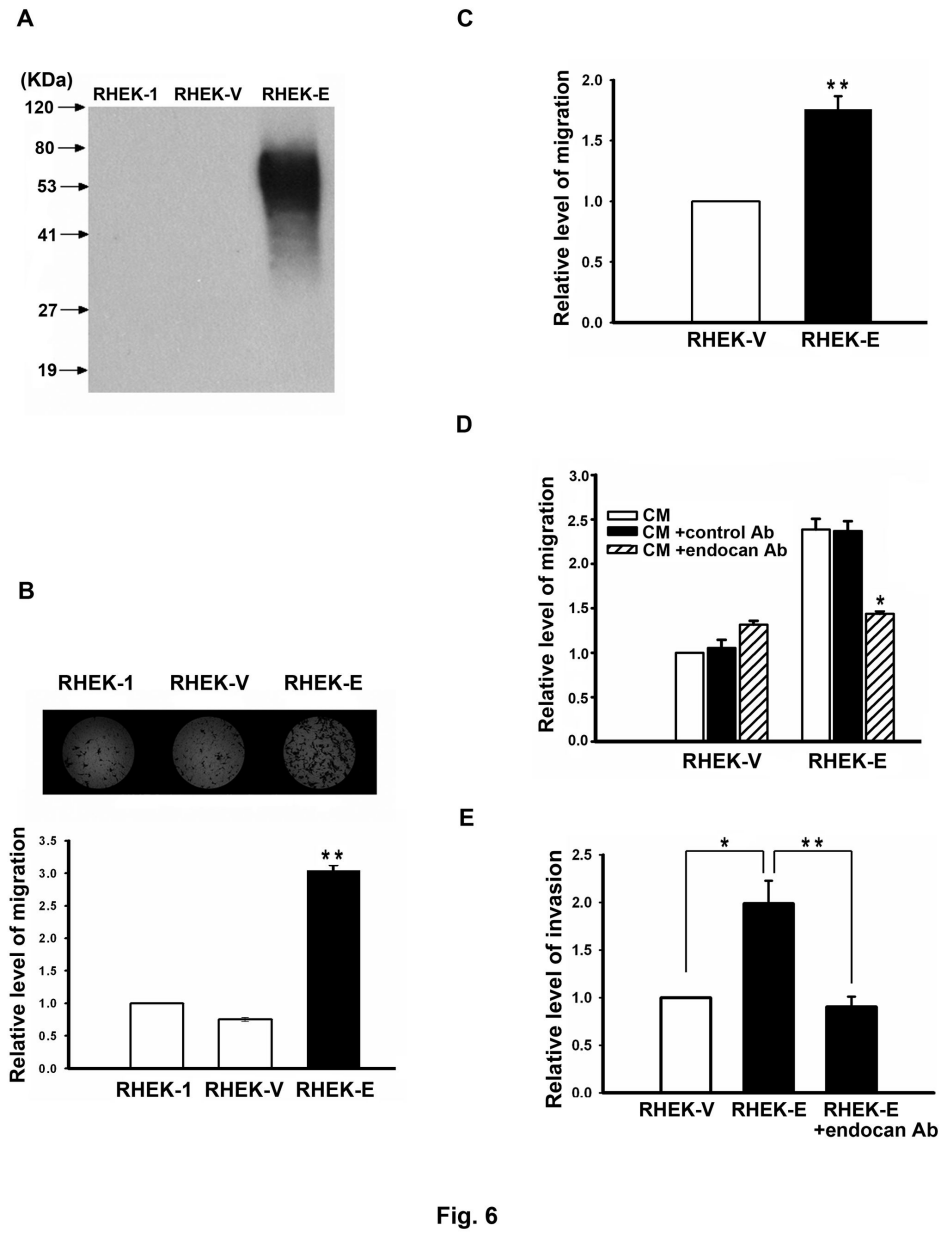
Endocan stimulates the migration and invasion ability of endothelial cells. (**A**) Western blot analysis shows the amount of endocan in the concentrated CM of RHEK-1, RHEK-Vec, and RHEK-endocan cells. (**B**) Effect of the CM from RHEK-1, RHEK-Vec, and RHEK-endocan cells on HMEC-1 cell migration. Upper panel, images of migrated HMEC-1 endothelial cells in the presence of CM from RHEK-1, RHEK-Vec, and RHEK-endocan cells. Lower panel, quantitation of migrated HMEC-1 endothelial cells. The migrated cells were counted in six randomly selected microscopic fields (x 200 magnification). Values represent means ± SD. ** *P* < 0.005 versus CM from RHEK-Vec cells. (**C**) Effect of the CM from RHEK-Vec and RHEK-endocan cells on HUVEC cell migration. The migrated cells were counted in six randomly selected microscopic fields. Values represent means ± SD. ** *P* < 0.005 versus CM from RHEK-Vec cells. (**D**) Pretreatment of CM from RHEK-endocan cells with anti-endocan antibody reduces its ability to induce HMEC-1 cell migration. CM from RHEK-Vec or RHEK-endocan cells was pre-treated with 200 ng/mL control or anti-endocan monoclonal antibody (Abnova) before using them for transmigration assays. The migrated cells were counted in six randomly selected microscopic fields. Values represent means ± SD. * *P* < 0.05 versus control antibody-pretreated CM. (**E**) Effect of endocan on the invasion ability of HMEC-1 endothelial cells. The cells that invaded and migrated to the lower chamber were counted in six randomly selected microscopic fields. Values represent means ± SD. * *P* < 0.05, ** *P* < 0.005. RHEK-V, RHEK-Vec cells; RHEK-E, RHEK-endocan cells.

Endocan is highly expressed in tumor endothelium [25-28] and is one of the genes involved in the switch from dormant to angiogenic tumors [37]. Moreover, endocan has been shown to be one of the tip cell-secreted molecules which may selectively act on stalk cells to regulate angiogenesis [52]. Together, these results suggest that endocan may play certain roles in angiogenesis. To investigate whether endocan had chemotactic activity to induce endothelial cell migration, we compared the chemotactic activity of conditioned medium (CM) from endocan-expressing RHEK-endocan cells with that of CM from vector-transfected RHEK-Vec cells or parental RHEK-1 cells by using the Boyden chamber transmigration assays. CM of RHEK-endocan cells contained abundant endocan while CM of RHEK-1 and RHEK-Vec cells contained undetectable amount of endocan (Figure 6A). Our transmigration assays indicated that CM from RHEK-endocan cells had higher ability to induce the migration of HMEC-1 endothelial cells (Figure 6B) and HUVEC (Figure 6C) than CM from RHEK-Vec or RHEK-1 cells, suggesting that endocan contained in the CM of RHEK-endocan cells may have chemotactic activity to induce endothelial cell migration. To confirm this, we pre-treated CM from RHEK-endocan or RHEK-Vec cells with anti-endocan antibody or control antibody before using them for transmigration assays. As shown in Figure 6D, while pre-treatment with control antibody did not affect the ability of CM from RHEK-endocan cells to induce endothelial cell migration, pre-treatment with anti-endocan antibody dramatically reduced the migration-inducing ability of CM from RHEK-endocan cells. These data clearly demonstrate that endocan has the chemotactic activity to induce endothelial cell migration. 

We also investigated whether endocan could affect the invasion ability of endothelial cells by using the Matrigel-coated Boyden chamber assays. As shown in Figure 6E, CM from RHEK-endocan cells had higher ability to induce HMEC-1 cells to invade through the Matrigel-coated filter than CM from RHEK-Vec cells. Moreover, pre-treatment of CM from RHEK-endocan cells with anti-endocan antibody could reduce its ability to induce endothelial cell invasion. Together, these data demonstrate that endocan can stimulate the invasion ability of endothelial cells. 

### The ability of endocan to induce endothelial cell migration is dependent on the glycan moiety and the phenylalanine-rich region of endocan

**Figure 7:**
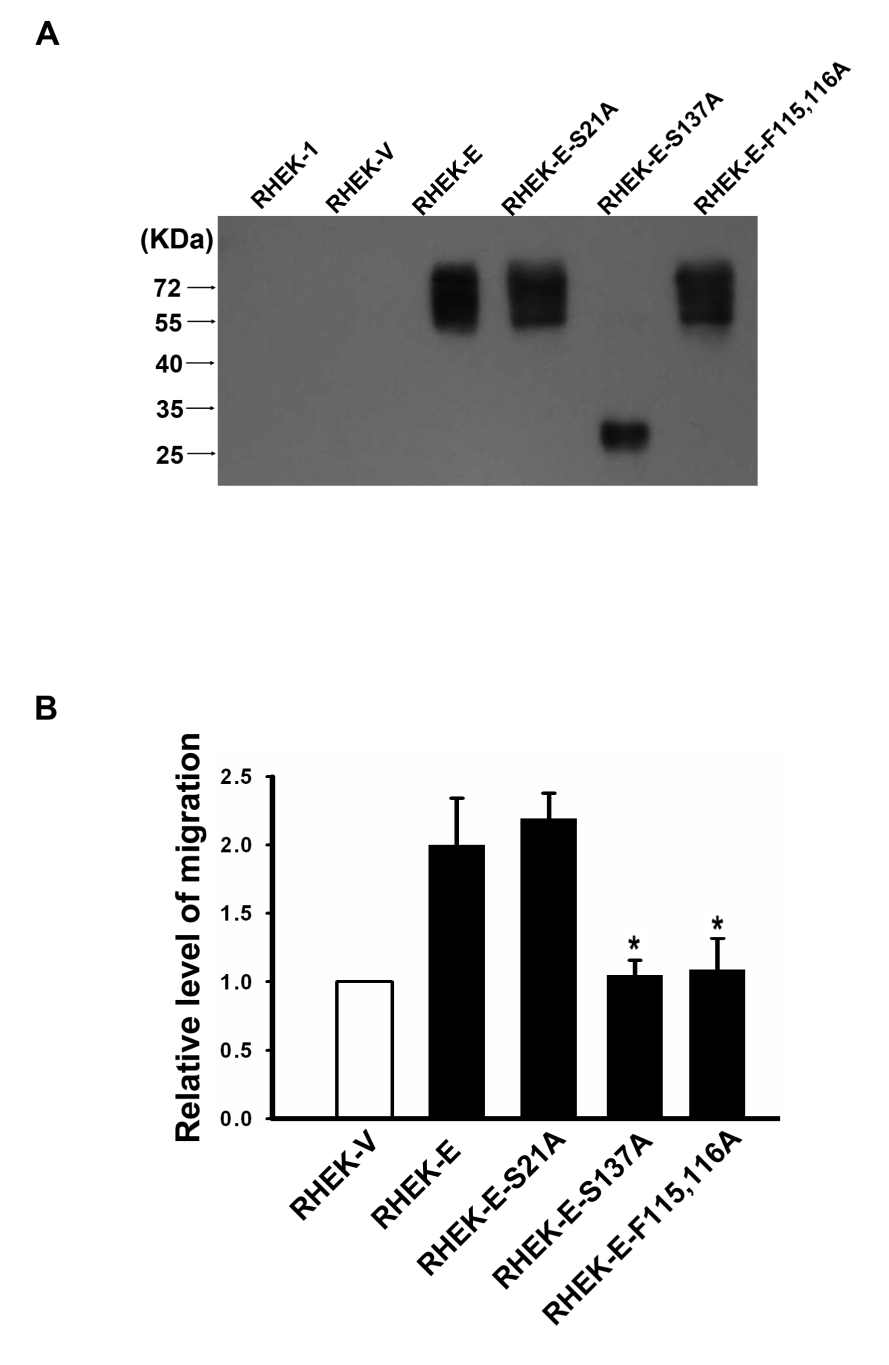
Glycan moiety and phenylalanine-rich region is required for endocan induction of endothelial cell migration. (**A**) Western blot analysis shows the expression of endocan in the concentrated CM of various RHEK-1-derived cell clones. (**B**) The chemotactic activity of CM from various RHEK-1-derived cell clones. The migrated HMEC-1 cells were counted in six randomly selected microscopic fields. Values represent means ± SD. * *P* < 0.05 versus CM from RHEK-endocan cells. RHEK-V, RHEK-Vec cells; RHEK-E, RHEK-endocan cells.

Both the glycan moiety and the phenylalanine-rich region of endocan have been shown to be required for the tumor-inducing activity of endocan [35]. To investigate whether the glycan moiety and the phenylalanine-rich region of endocan were also required for the chemotactic activity of endocan, we established pooled mutant endocan-expressing RHEK-1 cell clones (named RHEK-endocan-S21A, RHEK-endocan-F115,116A, and RHEK-endocan-S137A, respectively). The RHEK-endocan-S137A cells secrete nonglycanated mutant endocan (Figure 7A; [24,35]) while the RHEK-endocan-F115,116A and RHEK-endocan-S21A cells secrete fully glycanated mutant endocan (Figure 7A; [35]). Both endocan-S137A and endocan-F115,116A mutants have been shown to be defective in inducing tumors in *SCID* mice [35]. The endocan-S21A mutant was generated arbitrarily as a control. The concentration of endocan secreted to the CM by various RHEK-1-derived cell clones is shown in Table 3. The CM from various RHEK-1-derived cell clones were used in the Boyden chamber transmigration assays. To compare the chemotactic activity of CM from RHEK-endocan, RHEK-endocan-S21A, RHEK-endocan-S137A and RHEK-endocan-F115,116A cells, the concentration of endocan in the CM from these cells was adjust to similar level before using them for the transmigration assay. As shown in Figure 7B, while CM from RHEK-endocan-S21A cells had similar chemotactic activity to induce endothelial cell migration as CM from RHEK-endocan cells, CM from RHEK-endocan-S137A or RHEK-endocan-F115,116A had a much reduced chemotactic activity. These data demonstrate that the chemotactic activity of endocan is dependent on the glycan moiety and the phenylalanine-rich region of endocan. That glycan moiety is required for the chemotactic activity of endocan is also supported by our data that recombinant endocan purified from *E. coli* did not have ability to induce endothelial cell migration (data not shown). 

**Table 3:** Concentration of endocan in the conditioned medium of RHEK-1-derived cell clones.

Cell line	Secreted endocan (ng/mL) in CM
RHEK-1	34.6
RHEK-Vec	27.2
RHEK-Endocan	2343.0
RHEK-endocan-S21A	1360.5
RHEK-endocan-S137A	3162.0
RHEK-endocan-F115,116A	2322.2

## Discussion

Endocan has been shown to be upregulated in various human cancers, including non-small cell lung cancer [25], renal clear cell carcinoma [27,29,30], glioblastoma [26], heptocellular carcinoma [28,32], gastric cancer [31], pituitary adenoma [33], and bladder cancer [34]. Moreover, clinical studies have indicated that elevated expression of endocan is correlated with poor prognosis in lung, liver, gastric, bladder, and breast cancers [25,28,31,34,51]. NPC, an EBV-associated cancer, is one of the ten leading cancers in Taiwan. The expression of endocan in NPC is not known till now. In this study, by microarray analysis of gene expression, we found that endocan was one of the major genes upregulated by EBV LMP1 protein in RHEK-1 human epithelial cells. This induction of endocan by LMP1 was validated in several epithelial cell lines including an NPC cell line. To our knowledge, this is the first report showing that endocan expression can be regulated by a virus-encoded protein. In consistence with the above results, we found that endocan was overexpressed in about 52% of NPC specimens and its expression was correlated with expression of LMP1 protein in NPC tissues. Moreover, we demonstrated that endocan expression was associated with short survival in NPC patients and that endocan might be an independent prognostic factor for NPC. Together, these studies identify a new molecular marker that may predict the survival of NPC patients. 

Several lines of evidence suggest that LMP1 induction of endocan is the underlying cause of endocan expression in NPC. Firstly, our immunohistochemical staining data showed that the expressions of endocan and LMP1 were correlated in NPC tissues (Figure 4C). Secondly, LMP1 could upregulate endocan expression in the NPC cell line NPC-TW04 (Figure 2D). Thirdly, although endocan has been shown to be preferentially expressed in tumor endothelium in various cancers [25-28], our data indicated that endocan was expressed in both tumor endothelium and tumor cells in the NPC tissue (Figure 4A). The reason that endocan can be detected in NPC tumor cells is likely due to the presence of LMP1 in the NPC tumor cells. 

Our data indicate that CTAR1 and CTAR2 are the major functional domains of LMP1 involved in endocan induction, because mutant LMP1 with both CTAR1 and CTAR2 deleted almost completely lost its ability to induce endocan (Figure 3A). In consistence with this, we also found that LMP1 upregulated endocan expression through the NF-κB (activated by CTAR1 and CTAR2), MEK-ERK (activated by CTAR1), and JNK (activated by CTAR2) signaling pathways (Figure 3B). It has been shown that the NF-κB signaling pathway plays a positive role whereas the PI3K-AKT pathway plays a negative role in VEGF-mediated upregulation of endocan [26]. Our finding that LMP1-mediated upregulation of endocan was inhibited by BAY11-7082 (a specific inhibitor of the NF-κB pathway) but slightly enhanced by LY294002 (a specific inhibitor of PI3K) (Figure 3B) is consistent with the above result. Moreover, our finding that the MEK-ERK pathway was involved in induction of endocan is also consistent with the previous report [53].

One of the major findings of this study is that endocan expression is associated with poor prognosis in NPC (Figure 5A and Table 2). The molecular base for this association is not clear. However, several studies may point to the possible mechanisms underlying bad prognosis of NPC with endocan expression. Firstly, endocan has been shown to bind HGF/SF and enhance the HGF/SF-mediated mitogenic effect in human epithelial cells [24]. In many tumors, HGF/SF acts as a stroma-derived factor that bind to its receptor Met to promote proliferation, invasion and metastasis of cancer cells [54]. In NPC, HGF/SF is abundantly expressed in the interstitial tissues surrounding the tumor [55], whereas Met is expressed in LMP1-expressing NPC tumor cells [56]. Moreover, HGF/SF was shown to be able to enhance the metastatic potential of NPC tumor cells that express Met [57]. Take these results together with our finding of endocan expression in NPC tumor cells, it is possible that endocan secreted from NPC cells can enhance the effect of HGF/SF on the growth and metastasis of NPC. In this respect, it is of interest to note that the expression of endocan and Met is both correlated with the expression of LMP1 in NPC tumor cells (this study and ref [56].) and that the expression of Met has been shown to be associated with lymph node metastasis and bad prognosis of NPC [55,56]. Secondly, endocan has been shown to interact with LFA-1 and inhibit the interaction between LFA-1 and ICAM-1 [36]. LFA-1-ICAM-1 interaction is an important step for the firm adhesion of leukocytes to the endothelium, and thus could regulate the migration of leukocytes into tumor tissues. Moreover, LFA-1-ICAM-1 interaction has been implicated in the binding of cytotoxic lymphocytes and natural killer cells to tumor cells [58]. Through inhibiting LFA-1-ICAM-1 interaction, endocan secreted from NPC tumor cells may inhibit infiltration of leukocytes into tumor tissues and block the tumor-killing effect of cytotoxic lymphocytes and natural killer cells, leading to tumor development. Thirdly, endocan overexpression in nontumorigenic epithelial cells can induce tumor formation in *SCID* mice [35]. Moreover, recent studies indicated that endocan can promote cell survival, cell cycle progression, cell migration and invasion in hepatocellular carcinoma and colorectal cancer cells [32,59]. Together, these data indicate that endocan has tumorigenic activities. It is possible that endocan secreted by NPC tumor cells acts as an autocrine factor to promote tumor growth. Fourthly, we have found that endocan can stimulate endothelial cell migration and invasion and this ability of endocan is dependent on the dermatan sulfate side chain liked to it (Figure 6 and 7). Since dermatan sulfate is known to bind to a variety of signaling/adhesion molecules and growth factors [60], it is possible that endocan, through interacting with these cellular factors, can enhance angiogenesis in the NPC tumors.

LMP1 has been shown to be an independent prognostic factor for NPC [61]. Our data indicated that LMP1 expression was correlated with shorter survival in NPC patients (Figure 5B). However, by using univariate and multivariate Cox regression analysis, we found that endocan, but not LMP1, was an independent prognostic factor in the NPC patients (Table 2 and Table S3). Whether LMP1 is an independent prognostic factor for NPC is debatable. For instance, Kitagawa et al., recently reported that while Siah1 and HIF1α are independent prognostic factors for NPC, LMP1 is not [62].

Previous studies have suggested that endocan may play certain roles in regulation of angiogenesis (reviewed in ref [60].). However, strong evidence demonstrating that endocan plays a direct role in angiogenesis is lacking. Recently, Roudnicky et. al. showed that knockdown of endocan in HUVEC cells inhibits VEGF-A-induced tube formation and migration [34]. Brutsch et. al. showed that while knockdown of endocan inhibits capillary sprout formation, overexpression of endocan enhances sprouting in HUVEC cells [38]. Though these data suggest that endocan is involved in regulation of angiogenesis, they did not prove that endocan plays a direct role in angiogenic process. In this study, we showed that while the CM from endocan-expressing RHEK-1 cells could stimulate the migration and invasion ability of endothelial cells, anti-endocan antibody-pretreated CM could not (Figure 6), indicating that endocan can act as a chemotactic agent to induce endothelial cell migration and invasion. Furthermore, we demonstrated that the chemotactic activity of endocan was dependent on the glycan moiety and the phenylalanine-rich region of endocan (Figure 7). To our knowledge, this is the first report showing that endocan protein can act as a chemotactic agent to induce endothelial cell migration and invasion. 

In summary, we show for the first time that endocan is overexpressed in some NPC specimens and its expression is correlated with the expression of EBV LMP1 protein and with poor prognosis in NPC. We also show that LMP1 can upregulate the expression of endocan through the NF-κB, MEK-ERK, and JNK signaling pathways in epithelial cells and that endocan has chemotactic activity to induce endothelial cell migration and invasion. Since endocan has been shown to have several activities that may contribute to tumorigenesis, this study not only identifies a novel marker that may predict the survival of NPC patients but also provides a new insight to the tumorigenesis of NPC. 

## Supporting Information

Figure S1 **Western blot analysis showing that LMP1 can activate NF-kappaB, MEK-ERK, JNK, p-38 MAPK, and PI3K-Akt signaling pathways and that the inhibitors of these pathways are effective in inhibiting the respective signaling pathways.** RHEK/Tet-LMP1 cells were either untreated or treated with DMSO (solvent for inhibitors) or various signaling pathway inhibitors for 1 h. Cells were then either untreated or treated with 1 µg/mL doxycycline (Dox). After incubation for 36 h, cells were harvested and equal amounts of cell lysate were subjected to Western blot analysis. GAPDH serves as an internal control for amounts of protein loaded on the gel.
(PDF)

Figure S2 **Immunohistochemical staining of vWF in NPC tissues.** Rabbit polyclonal antibody against vWF (purchased from DAKO) was used as primary antibody. The sections were color-developed with Super Sensitive Polymer-HRP IHC Detection System (BioGenex, San Ramon, CA, USA). The sections were counterstained with hematoxylin. The brown color represents vWF staining. Arrowheads indicate vascular endothelial cells. T, tumor cells.
(PDF)

Figure S3 **Immunohistochemical staining of endocan in NPC tissues.** Mouse monoclonal antibody against endocan (purchased from Abnova) was used as primary antibody. The sections were color-developed with Super Sensitive Polymer-HRP IHC Detection System (BioGenex, San Ramon, CA, USA). The sections were counterstained with hematoxylin. The brown color represents endocan staining. Arrowheads indicate vascular endothelial cells. T, tumor cells.
(PDF)

Methods S1 **Supplementary methods-cDNA microarray analysis.**
(DOC)

Table S1 **Real-time PCR primers.**
(PDF)

Table S2 **Microarray analysis of LMP1-regulated genes.**
(XLS)

Table S3 **Univariate and multivariate Cox regression analysis indicating that LMP1 is not an independent prognostic factor for NPC.**
(PDF)
