# Supplementary material for: Upregulation of Endocan by Epstein-Barr Virus Latent Membrane Protein 1 and Its Clinical Significance in Nasopharyngeal Carcinoma
Source: PLoS One. 2013 Dec 5;8(12):e82254. doi: 10.1371/journal.pone.0082254 (PMC3855342; doi:10.1371/journal.pone.0082254)
Supplement: Figure S1 — Western blot analysis showing that LMP1 can activate NF-kappaB, MEK-ERK, JNK, p-38 MAPK, and PI3K-Akt signaling pathways and that the inhibitors of these pathways are effective in inhibiting the respective signaling pathways. RHEK/Tet-LMP1 cells were either untreated or treated with DMSO (solvent for inhibitors) or various signaling pathway inhibitors for 1 h. Cells were then either untreated or treated with 1 µg/mL doxycycline (Dox). After incubation for 36 h, cells were harvested and equal amounts of cell lysate were subjected to Western blot analysis. GAPDH serves as an internal control for amounts of protein loaded on the gel. (PDF) [file pone.0082254.s001.pdf]

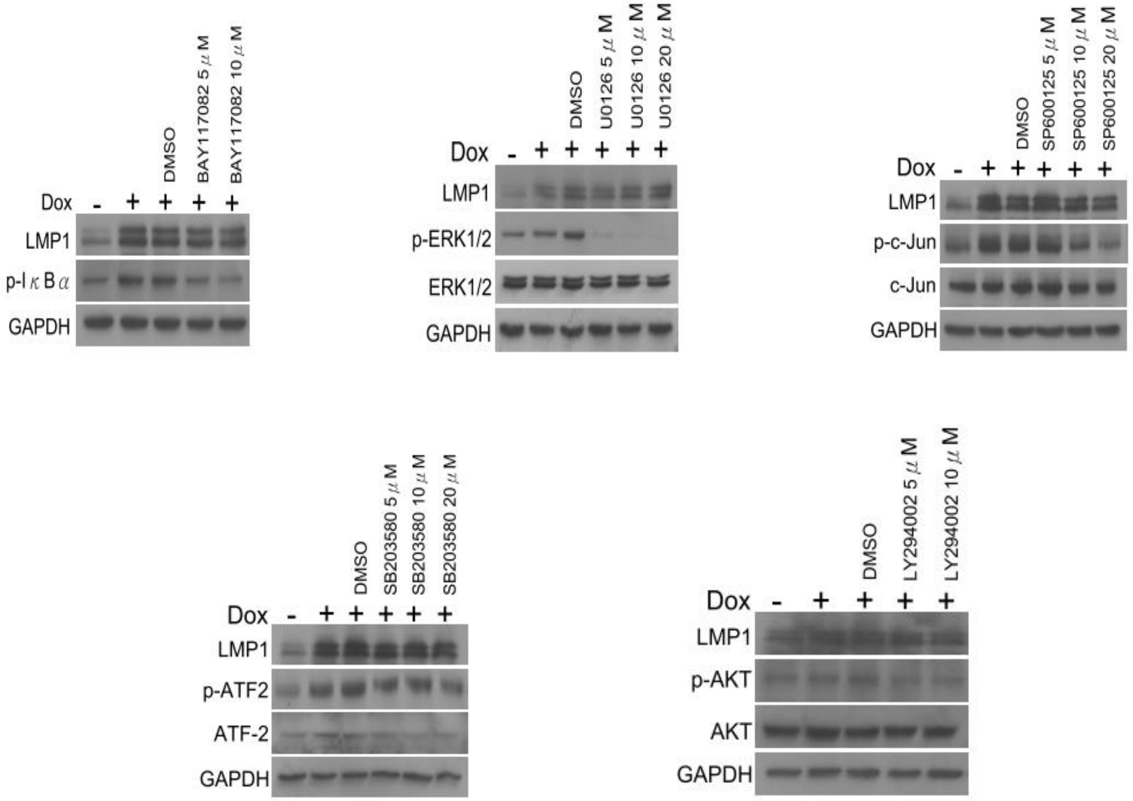

Figure S1. Western blot analysis showing that LMP1 can activate the NF-κB, MEK-ERK, JNK, p38 MAPK, and PI3K-Akt signaling pathways and that the inhibitors of these pathways are effective in inhibiting the respective signaling pathways. RHEK/Tet-LMP1 cells were either untreated or treated with DMSO (solvent for inhibitors) or various signaling pathway inhibitors for 1 h. Cells were then either untreated or treated with 1 μg/mL doxycycline (Dox). After incubation for 36 h, cells were harvested and equal amounts of cell lysate were subjected to Western blot analysis. GAPDH serves as an internal control for amounts of protein loaded on the gel.
