# Supplementary material for: Upregulation of Endocan by Epstein-Barr Virus Latent Membrane Protein 1 and Its Clinical Significance in Nasopharyngeal Carcinoma
Source: PLoS One. 2013 Dec 5;8(12):e82254. doi: 10.1371/journal.pone.0082254 (PMC3855342; doi:10.1371/journal.pone.0082254)
Supplement: Figure S3 — Immunohistochemical staining of endocan in NPC tissues. Mouse monoclonal antibody against endocan (purchased from Abnova) was used as primary antibody. The sections were color-developed with Super Sensitive Polymer-HRP IHC Detection System (BioGenex, San Ramon, CA, USA). The sections were counterstained with hematoxylin. The brown color represents endocan staining. Arrowheads indicate vascular endothelial cells. T, tumor cells. (PDF) [file pone.0082254.s003.pdf]

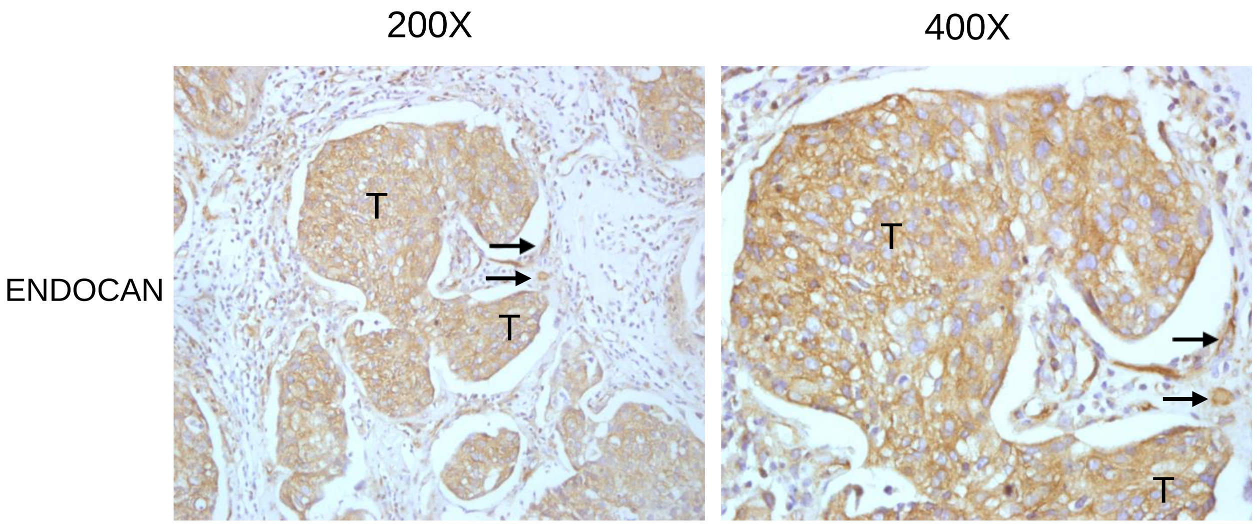

Figure S3. Immunohistochemical staining of endocan in NPC tissues. Mouse monoclonal antibody against endocan (purchased from Abnova) was used as primary antibody. The sections were color-developed with Super Sensitive Polymer-HRP IHC Detection System (BioGenex, San Ramon, CA, USA). The sections were counterstained with hematoxylin. The brown color represents endocan staining. Arrowheads indicate vascular endothelial cells. T, tumor cells.
