# Supplementary material for: Upregulation of Endocan by Epstein-Barr Virus Latent Membrane Protein 1 and Its Clinical Significance in Nasopharyngeal Carcinoma
Source: PLoS One. 2013 Dec 5;8(12):e82254. doi: 10.1371/journal.pone.0082254 (PMC3855342; doi:10.1371/journal.pone.0082254)
Supplement: Methods S1 — Supplementary methods-cDNA microarray analysis. (DOC) [file pone.0082254.s004.doc]

**Supplementary Methods**

**cDNA microarray analysis**

Human UniversoChip 8K cDNA array (Asia BioInnovations Corporation, Taipei, Taiwan), containing 7597 genes, was used in this study. Total RNA from doxycycline-treated or untreated RHEK/Tet-LMP1 cells was extracted by TRIzol reagent (Invitrogen, Carlsbad, CA). mRNA was isolated from total RNA with a Dynal MPC-s kit (Dynal Biotech, Lake Success, NY). For synthesis of microarray probes, mRNA from doxycycline-treated or untreated RHEK/Tet-LMP1 cells was reverse transcribed with Superscript II RNase H-reverse transcriptase (Life Technologies) in the presence of Cy5-dUTP and Cy3-dUTP (Amersham Inc., Piscataway, NJ), respectively. Labeled cDNA was purified and resuspended in the hybridization buffer as described (Methods Enzymol 303: 179-205). Equivalent amounts of labeled cDNA were hybridized to the arrays overnight at 70 ℃. The arrays were washed as described previously (Methods Enzymol 303: 179-205). Hybridized slides were scanned using the GenePix 4000B scanner (Axon Instrument, Union City, CA), and images were processed using the GenePix Pro 3.0 (Axon Instrument). Microarray data were analyzed using the eGenomix V1.0 (Asia BioInnovations Corporation) and EXCEL (Microsoft, Seattle, WA) software.
