# Supplementary material for: Upregulation of Endocan by Epstein-Barr Virus Latent Membrane Protein 1 and Its Clinical Significance in Nasopharyngeal Carcinoma
Source: PLoS One. 2013 Dec 5;8(12):e82254. doi: 10.1371/journal.pone.0082254 (PMC3855342; doi:10.1371/journal.pone.0082254)
Supplement: Table S1 — Real-time PCR primers. (PDF) [file pone.0082254.s005.pdf]

Table S1. Real-time PCR primers.

Gene symbol	forward	reverse
MMP9	GACGCAGACATCGTCATCCA	AACTCGTCATCGTCGAAATGG
E2F1	TCCTGAGACCCAGCTCCAAG	AGAAGTGACCTCCTGGGATGG
ENDOCAN	AGGCATGGATGGCATGAAGT	GCCTGACTGGCAGTTGCAG
GADD45G	AAAGCACAGCCAGGATGCAG	GCAGCCAGCACACAGAAGG
HOXD3	CTGTGCCACTGCAGGAGAGA	CGGCACAAGTAGCGGTTGA
BMP6	TTCCCACTCAACGCACACAT	GGTGAACCAAGGTCTGCACAA
NOS2A	GTGGCCCACACCCTGAAG	TCGACCTGCTCCTCATTCAATT
RAD52	TTGGAGGACGTGACAGCCA	CTCTGCCTCAGGGCCTTCT
RASA1	CGGGACAGTGGACGAAGGT	TGCCTGAGGCGTTCTTCTG
IGF1R	TCCTGACATGCTGTTTGAACTGA	CAGCTTGTTCTCCTCGCTGTAGT
ITGB8	TTGGGTTGCTTAAAGTCCTGATC	CGTCGGTAGGTGACTGCTCTT
MMP10	TTTGGCCCTCTCTTCCATCATAT	AAACGGTGTCCCTGCTGTTAAC
MET	CCCGAATACTGCCCAGACC	GATATCCGGGACACCAGTTCA
GAPDH	GAAGGTGAAGGTCGGAGTC	GAAGATGGTGATGGGATTTC
