# Supplementary material for: Upregulation of Endocan by Epstein-Barr Virus Latent Membrane Protein 1 and Its Clinical Significance in Nasopharyngeal Carcinoma
Source: PLoS One. 2013 Dec 5;8(12):e82254. doi: 10.1371/journal.pone.0082254 (PMC3855342; doi:10.1371/journal.pone.0082254)
Supplement: Table S3 — Univariate and multivariate Cox regression analysis indicating that LMP1 is not an independent prognostic factor for NPC. (PDF) [file pone.0082254.s007.pdf]

Table S3. Univariate and multivariate Cox regression analysis indicating that LMP1 is not an independent prognostic factor for NPC.

Characteristics		Univariate analysis		Multivariate analysis	
	**No. patients**	***P***	**Harzard ratio(95% CI**[**]**)**	***P***	**Hazard ratio(95% CI)**
Age[*]	36	0.046	1.038 (1.001-1.076)	0.025	1.053 (1.007-1.101)
Sex					
Male	28	0.778	1.171 (0.391-3.506)	0.634	1.318 (0.423-4.109)
Female	8				
Histologic classification					
Type II	9	0.150	0.405 (0.118-1.388)	0.069	0.269 (0.065-1.107)
Type III	27				
Distant metastasis					
Presence	8	0.008	3.566 (1.386-9.176)	0.206	1.947 (0.694-5.465)
Absence	28				
LMP1					
Positive	17	0.081	2.232 (0.907-5.493)	0.161	1.955 (0.765-4.994)
Negative	19				

[*]Age was treated as a continuous variable

[**]CI, confidence interval
